# Effects of fasting during Ramadan on cerebrovascular hemodynamics: A transcranial Doppler study

**Published:** 2016-01-05

**Authors:** Masoud Mehrpour, Fahimeh H. Akhoundi, Zahra Rezaei

**Affiliations:** 1 Department of Neurology, Firoozgar Hospital, Iran University of Medical Sciences, Tehran, Iran; 2 Firoozgar Clinical Research Development Center (FCRDC), Iran University of Medical Sciences, Tehran, Iran

**Keywords:** Fasting, Cerebrovascular Circulation, Transcranial Doppler, Blood Flow Velocity

## Abstract

**Background:** To determine whether Islamic fasting would change cerebral blood flow during Ramadan.

**Methods:** The study group comprised 20 subjects (16 males and 4 females) on a regimen of 1 month food and water intake restriction, according to Islamic fasting ritual. Subjects were evaluated for cerebral bolo flow through a middle cerebral artery (MCA) by means of transcranial Doppler (TCD), the day before starting Ramadan fasting and the day after the month of Ramadan.

**Results:** Our results showed no statistically significant changes after Ramadan in cerebrovascular hemodynamic, in comparison before Ramadan.

**Conclusion:** Although some studies showed metabolic changes during Ramadan fasting (increasing hematocrite, decreasing amount of hemoglobin, dehydration, platelet aggregation, and lipid profile alternations) the findings suggest that Islamic fasting has no significant effects on cerebral blood flow.

## Introduction

The Islamic ritual of fasting during the month of Ramadan requires Muslim to abstain from eating, drinking, and smoking during the hours of daylight.

During the daylight hours of Ramadan fasters undoubtedly get dehydrated. Several measures have been used to give estimates of hydration status of individuals, including a significant increase in hematocrite, serum albumin and serum creatinine indicating dehydration due to water deprivation.^1^

Ramadan fasting led to decrease in the platelet aggregation factors [adenosine 5-diphosphate (ADP) and collagen] it also causes an increase in bleeding time (BT) and coagulation time.^2^

Hence, we decided to determine whether Islamic fasting which causes hemodynamic and metabolic blood changes has any significant effect on brain hemodynamics. According to the literature review most of the previous studies either evaluated metabolic alterations due to fasting or blood components fluctuation furthermore, none of them have decelerated fasting effects on brain hemodynamic. Thus, our study was designed to perform this evaluation in fasters. We aimed to measure the impact of blood alternations due to fasting on cerebrovascular hemodynamics through middle cerebral artery (MCA) velocity by means of transcranial Doppler (TCD). We hypothesized that fasting will result in decreased MCA peak systolic velocity (PSV), pulsatility index (PI) and resistance index (RI) by two mechanisms: a decrease in hematocrit and an improved lipid profile, which are immediate and late effects of fasting, respectively.

## Materials and Methods

An observational study was performed in Firoozgar Academic Hospital affiliated with Iran University of Medical Sciences (IUMS), Tehran, Iran, between June 2012 and July 2012. The study protocol was approved by Research Committee of IUMS, and the ethical standards set out in the Helsinki Declaration of 1975.

Duration of fasting was about 16 hour, with average climate of 36.4 °C and 40.0% humidity in Tehran. The mean number of consecutive fasting was 28 ± 2 days. A total number of 20 volunteer, (16 male and 4 female) with a mean age of 33.50 ± 9.16 year (range: 21-55 year). Inclusion criteria included healthy persons who indicated that they were going to fast during Ramadan, with general good health and no taking medications for chronic disease. Exclusion criteria included any chronic or acute disease, taking medications, cigarette smoking. Furthermore, none of the female subjects were pregnant or using contraceptive pills.

A consent form was taken from all the recruited cases and referred to the Neurology Laboratory of Firoozgar Hospital. After recruitment of participants a day before beginning Ramadan, and the day after finishing Ramadan, TCD was performed for all of the cases to measure blood flow velocity of both right and left MCAs. All the TCD measurements were performed by the same experienced neurologist. Cerebral blood flow was estimated by 2 MHz TCD ultrasound probe (Atys-Looki, France).

The probe fixed over the temporal window to insonate the proximal segment of MCA (M1). Once the optimal signal-to-noise ratio was obtained, the probe was covered with an adhesive ultrasonic gel and secured with a headband device (Multigon) to maintain optimal insonation position. Optimization of the Doppler signals from the MCA was performed by varying the sample volume depth in incremental steps and at each depth, varying the angle of insonance to obtain the best-quality signals from the Doppler frequency. The depth of recorded parameters was the same in pre and post TCD measurements. Both right and left MCAs velocities were monitored reporting the main indexes including PSV, end diastolic velocity (EDV). Consequently, other indexes such as PI and RI were calculated using the following formulas:

PI = (PSV-EDV)/Mean flow volume

RI = (PSV-EDV)/PSV

Qualitative and quantitative variables were described by frequency percentages, mean and standard deviation (SD), respectively. Collected data were subjected to statistical analysis using SPSS software (version 16, SPSS Inc., Chicago, IL, USA). Data distribution was assessed by the Kolmogorov-Smirnov test in all groups; the Mann-Whitney U-test and paired sample t-test were used on occasion, to evaluate the statistical significance of differences before and after Ramadan. A P < 0.050 was considered to be significant. Data were presented as mean ± SD [95% confidence interval (CI)].

## Results

About 20 healthy volunteers male/female (80/20) the mean age of subjects was 33.5 (range 21-55 years, SD = 9.16). None of demographic, baseline was significantly different between our subjects except for age (P < 0.001). All data were normally distributed in each group according to the Kolmogorov-Smirnov test.

**Figure 1:**
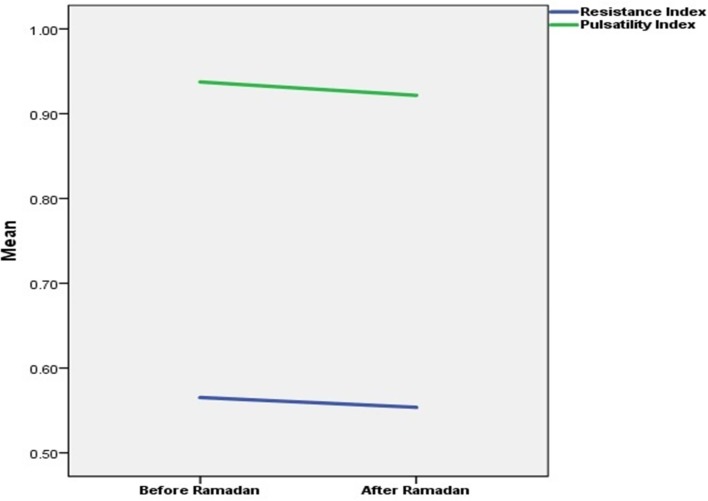
Resistance and pulsatility indices before and after Ramadan

TCD findings of right and left MCAs were compared between before and after Ramadan fasting, PSV, EDV, PI, RI were all similar before and after fasting (Tables 1 and 2). Hence, there were no significant correlation between velocity indices before and after Ramadan fasting [the mean (95% CI), Figure 1].

## Discussion

To our knowledge, there are no reports on the effect of Islamic fasting on cerebrovascular hemodynamic. In this study, Ramadan fasting caused no significant fluctuations on brain blood flow velocity indices, which is in favor of low risk for cerebrovascular events.

**Table 1:** Comparison of transcranial Doppler findings of left middle cerebral artery between before and after Ramadan

**Velocity index**	**Before Ramadan**	**After Ramadan**	**P**
PSV (cm/s)	79.70 ± 13.38	78.10 ± 13.62	0.570
Range	(51-104)	(56-106)	
EDV (cm/s)	34.45 ± 9.20	39.85 ± 9.90	0.610
Range	(24-69)	(21-65)	
RI	0.56 ± 0.70	0.55 ± 0.92	0.490
Range	(0.33-0.67)	(0.26-0.66)	
PI	0.94 ± 0.20	0.55 ± 0.92	0.830
Range	(0.62-1.60)	(0.26-0.66)	

PSV: Peak systolic velocity; EDV: End diastolic velocity; RI: Resistance index; PI: Pulsatility index

**Table 2:** Comparison of transcranial Doppler findings of right middle cerebral artery‎ between before and after Ramadan

**Velocity index**	**Before Ramadan**	**After Ramadan**	**P**	
PSV (cm/s)	78.25 ± 13.58	83.65 ± 16.65		0.530
Range	(0.59-1.05)	(58-125)		
EDV (cm/s)	34.40 ± 8.71	35.50 ± 8.62		0.590
Range	(0.24-0.69)	(24-58)		
RI	0.55 ± 0.60	0.59 ± 0.95		0.430
Range	(0.39-0.64)	(0.47-0.95)		
PI	0.91 ± 0.11	0.93 ± 15.00		0.850
Range	(0.70-1.10)	(0.59-1.15)		

PSV: Peak systolic velocity; EDV: End diastolic velocity; PI: Pulsatility index; RI: Resistance index

The variability in daily fasting time is one of several confounding variables that influence the effect of Ramadan fasting on health-related biomarkers. The period in which the person fasts may vary depending on the geographical location of the country and the season of the year, and can be as long as 18 hours/day in the summer of temperate regions. This seasonal shift dramatically impacts the amount of daily fasting time that occurs in any given location, but the average time is 12 hours in length.^3^^-^^5^

Ramadan fasting resulted in significant weight loss, however, most of the weight loss will be regained during 2 weeks after Ramadan. The basis for the potential effects of Ramadan fasting on different biochemical parameters has been found in investigations which reports various metabolic effects that have been produced either by a decrease in meal frequency.^6^^-^^9^

A previous investigation compares pre and post Ramadan lipid profile and lipoproteins. Lipid profile including plasma total cholesterol, low-density lipoprotein (LDL), high-density lipoprotein (HDL) and triglyceride (TG) were measured, these parameters revealed a significant reduction in energy intake, plasma total cholesterol, LDL and TG levels toward the end of Ramadan in fasting group despite non-fasting group, indeed impact of Ramadan on improving lipid profile may have positive effect on atherosclerosis incidence.^10^^-^^15^

Another recent study by Mirghani et al., declerated the effect of maternal fasting on uterine blood flow during midtrimester (by means of Doppler flow velocitometry). Both uterine arteries velocity indexes (PSV, EDV, RI, PI) were similar in both case and control groups. It shows that maternal fasting is not associated with significant changes in uterine artery Doppler flow velocitometry.^16^

During the daylight hours of Ramadan fasting practicing probably causes dehydration by the mass of body. Water minus the amount of metabolic water that is produced over this period.

According to the literature review, studies show that Ramadan fasting does not affect the overnight urine volume or osmolarity but sample collected during afternoon were very high in level of osmolarity and showed a decrease in urine volume. No detrimental effects on health have as yet been directly attributed to intermittent water negative balance (dehydration during light hours and water compensation during night).^17^

Another study shows, in healthy persons, fasting Ramadan does not induce abnormalities of urinary volume, osmolarity, pH, solute, and electrolyte excretion. Changing urea and creatinin are usually insignificant, and sodium and potassium fluctuations are negligible.^18^

Ramadan fasting leads to decrease in the platelet aggregating agents (e.g. ADP and collagen) in vitro and also causes an increase in BT and clotting time which shows platelet activation, but these changes remained within physiological limits, however, platelet count did not change during fasting.^2^

Compared to the previous study, it has been reported hematological parameters such as hemoglobin, pack cell volume^19^ and count of erythrocyte, showed a significant decrease during Ramadan which resulted in an increase in anemia prevalence, in order to avoid iron deficiency anemia, the consumption of iron-rich food is recommended.

The burdens of excess weight and hypertension may be alleviated, and it could be beneficial in the long-term by reducing cardiovascular risk.^20^

Although our study has some limitations including small sample size and considering short-term effect of fasting, it must be taken to account that our investigation about the effect of fasting on cerebrovascular alternations were done for the first time, and it was not reported before. In conclusion, Ramadan fasting conditions in healthy persons does not effect cerebrovascular hemodynamic. It is strongly recommended to evaluate the brain changes during fasting in larger sample size and by means of other techniques such as duplex and neuroimaging, and also it is important to consider climate circumstances in which fasting is done.

## Conclusion

The results of our study showed that 1 month of daily fasting has no statistically significant effect on cerebral circulation indices including PSV, EDV, PI, and RI. To our knowledge, this is the first study evaluating the effects of fasting on cerebrovascular circulation. Future studies are needed to examine the reproducibility of our results in elder populations.

## Notes:

**How to cite this article:** Mehrpour M, Akhoundi FH, Rezaei Z. Effects of fasting during Ramadan on cerebrovascular hemodynamics: A transcranial Doppler study. Iran J Neurol 2016; 15(1): 23-7.
